# The COVID-19 infodemic in Brazil: trends in Google search data

**DOI:** 10.7717/peerj.13747

**Published:** 2022-08-04

**Authors:** Maria da Penha Harb, Lena Veiga e Silva, Nandamudi Vijaykumar, Marcelino Silva da Silva, Carlos Renato Lisboa Frances

**Affiliations:** 1 Institute of Technology, Federal University of Pará, Belém, Pará, Brazil; 2 University of Amazon, Belém, Pará, Brazil; 3 National Institute for Space Research, São José dos Campos, São Paulo, Brazil; 4 Federal University of Western Pará, Santarém, Pará, Brazil

**Keywords:** COVID-19, Infodemic, Google Trends, Brazil, Denialism, Clustering

## Abstract

**Background:**

Since the beginning of the new coronavirus pandemic, there has been much information about the disease and the virus has been in the spotlight, shared and commented upon on the Internet. However, much of this information is infodemics and can interfere with the advancement of the disease and that way that populations act. Thus, Brazil is a country that requires attention, as despite the fact that in almost two years of pandemic it has shown a devastating numbers of deaths and number of cases, and generates false, distorted and malicious news about the pandemic. This work intends to understand the attitudes of the Brazilian population using infodemic queries from the Google Trends search tool and social and income variables.

**Methods:**

Data from infodemic research carried out on Google Trends, between January 1, 2020 and June 30, 2021, with socioeconomic data, such as income and education, were unified in a single database: standardization and exploratory and multivalued techniques based on grouping were used in the study.

**Results:**

In the analysis of the search trend of infodemic terms, it is clear that the categories of Prevention and Beliefs should stand out in Brazil, where there is a diverse culture. It is followed by the COVID-19 Treatment category, with treatments that were not those recommended by the authorities. Income transfer programs and information on socioeconomic variables did not have much impact on infodemic surveys, but it was observed that states where President Bolsonaro has more supporters had researched more infodemic information.

**Conclusions:**

In a country as geographically large as Brazil, it is important that political authorities go to great lengths to disseminate reliable information and monitor the infodemic in the media and on the internet. It was concluded that the denial of the pandemic and the influence of political leaders influenced the search for infodemic information, contributing to a disorganization in the control of the disease and prevention measures.

## Introduction

In December 2019, a new virus from the coronavirus family (called SARS-CoV-2) was identified in China in humans. The virus was discovered in Wuhan and, on January 23, 2020, driving the city into isolation and making it the epicenter of the epidemic (Guo et al., 2020). The World Health Organization (WHO) decided to formally declare the COVID-19 as a pandemic after the number of cases had increased very quickly, affecting each and every country. COVID-19 was announced as a new coronavirus-based disease (World Health Organization, 2020a).

National health bodies and the WHO started to disseminate reliable information to fight fake news. Handbooks and guidance procedures were prepared and distributed. However, to reach a large scale of the population, the use of the Internet is paramount. Digital healthcare solutions have become a promising option to tackle and fight the outbreak in modern times. Several websites published information about COVID-19 and issued different instructions to its users on how to prevent the spread of the virus, such as keeping distance between them, wearing masks, and washing hands (Hernández-García & Giménez-Júlvez, 2020).

With the very significant increased use of computers, tablets and smartphones, an opportunity is created for the rapid dissemination of information *via* the Internet and social media. Unfortunately, there is no guarantee that this dissemination is correct (Oyeyemi, Gabarron & Wynn, 2014). As in previous epidemics, Ebola or Zika infections, the Internet has become one of the favorite mechanisms for spreading misinformation (Oyeyemi, Gabarron & Wynn, 2014; Venkatraman et al., 2016). This has implications for public health behavior and health-related decision-making (Gesser-Edelsburg et al., 2018).

Both the impact of the disease, which was already large, and the lack of information associated with it allowed misinformation to appear quickly and spread across various social media platforms (Kouzy et al., 2020). In this context, on February 15, 2020, WHO Director Tedros Adhanom Ghebreyesus at the Munich security conference said, “we are not just fighting an epidemic, we are fighting an infodemic” (World Health Organization, 2020b). There has been an excess of information, some accurate and some not, which makes it difficult to find reputable sources and reliable guidance when needed. It is known that fake news spreads faster and easier than the virus (new coronavirus) and is equally dangerous (Organização Pan-Americana de Saúde, 2020).

### The pandemic and infodemic in Brazil

The first case of COVID-19 recorded in Brazil was in the city of São Paulo (SP) on February 26, 2020 (shortly after the first case in China), soon after with local transmissions, with the first death on March 17th. Even before the confirmation of the first case, on February 3, the country declared a Public Health Emergency of National Importance and, on March 20, the National Congress recognized the State of Public Calamity, valid until December 31 of this year (Ministério da Saúde, 2021b). Since March 2020, many Brazilian states have adopted isolation and social distancing measures, including the adoption of lockdown measures. Measures of this nature allowed time for organizing health care resources and epidemiological surveillance, to control COVID-19 (Aquino et al., 2020).

However, because Brazil is a territorially large country and has social and income differences, the pandemic has progressed differently in different regions. Besides the deficiency in health resources, the Government was not of great help as they announced contradictory data with respect not only to the implications of the pandemic (Silva et al., 2020), but also unconventional forms for preventing and treating the disease. In addition, there were websites with fake or questionable content.

False news that has been shared on the Internet can be extremely harmful to the control and prevention of the pandemic and the spread of the virus, such as (Ministério da Saúde, 2021b): Flu vaccine increases the risk of getting sick from coronavirus; masks without quality distributed by the Brazilian Ministry of Health; China’s donation masks are contaminated with coronavirus; coffee prevents coronavirus; alkaline foods prevent coronaviruses; lemon tea with hot baking soda cures coronavirus.

According to the United Nations Educational, Scientific and Cultural Organization, the impacts of disinformation related to the pandemic are deadlier than disinformation on other subjects, such as politics and democracy (UNESCO, 2020). To combat the creation and dissemination of fake news, the Brazilian Senate approved, on June 30, 2020, the bill 2630: the Brazilian Law on Freedom, Responsibility and Transparency on the Internet, in which one of the items is to combat the dissemination of fake information on the Internet (Senado Notícias, 2020).

Brazil is the largest country in South America, geographically and in terms of population. Understanding the behavior of the population can highly influence and help authorities to prevent false information and news from circulating through digital media and social networks, thus interfering with transmission, and combating the pandemic, inside and outside the country.

In this respect of combat and control, health policy authorities have begun to identify Internet search engines as potential indicators for surveillance and health, such as the Google Trends (GT), a repository of publicly available information on user research in real time and patterns (Arora, Mckee & Stuckler, 2019).

### Information search and denial

The Internet is revolutionizing the way epidemic intelligence is collected and offers solutions to some of these challenges. Freely available sources of information can allow us to detect disease outbreaks earlier with reduced cost and greater transparency in reporting (Wilson & Brownstein, 2009).

Thus, at the beginning of the pandemic, with the increase in the dissemination of SARS-CoV-2 in Brazil, there was a great interest of the population for considerable search about the disease. GT report (Google Trends, 2021a), in Brazil, points out that web queries for the infodemic term “coronavirus” increased considerably, reaching its peak on March 15 and 21, 2020. Among other infodemic terms, there was a search for the virus name, origin, and treatment. Many websites were created with the objective of sharing false information leading the Brazilian population to an incorrect understanding. Recent research also points out that the media (Alvarez, Etxebarrieta & Santamaría, 2021) and even scientists have contributed to the infodemic phenomenon, including stigma and the attribution of wrong denominations (Rovetta & Castaldo, 2022).

Understanding the situation at risk, health authorities started disseminating booklets and guidelines to warn not only about the fake news but also the inability of controlling their dissemination. The Ministry of Health website (Governo do Brasil, 2021) created a web page in order to understand what false, doubtful, misleading news are and to suggest what to do to check the content of such news.

Apprehension about malicious, dubious and false news exceed 80% according to an annual report by the Digital News Report (2021). This factors linked to sociocultural questions and the way Brazilians perceive science and approach health and denial (Collucci, 2021). According to researcher Mariana Silva, cited in Collucci (2021), data analysis shows that the President, by advocating the so-called early treatment (COVID kit), manages to gain support among people who are not his loyal followers because the speech is allied to a popular culture very attached to medicine, where the population needs to be medicated even without scientific proof.

Rathsam (2021) proposed that a large part of the population has adopted denialism about the situation of the pandemic in the country. The president never provided attention to the seriousness of the disease. He has also shown contempt for the preventive measures as well as allowed the underreporting of epidemiological data. The government did not look into healthcare strategy to control the outbreak and suggested therapeutic treatments with no scientific evidence. His denial towards the pandemic led him to also discredit vaccines. This scenario led the population into uncertainties in embracing preventive protocols which ended up implicating the fight against the disease besides threatening the democracy.

### Present study

The present work investigates infodemic searches in Brazilian states from January 1, 2020 to June 30, 2021. GT (Google Trends, 2021b) was the tool employed to provide insights as well as potential indicators of changes in information patterns of the COVID-19 pandemic in Brazil. In addition to this information, data were also obtained from six more Brazilian government portal databases.

Based on all the data, infodemic scenarios were compiled for Brazil. An analysis of research trends on infodemic terms were performed dividing into five categories on pandemic issues. An analysis related to the country’s social and political scenarios, with exploratory and cluster-based multivariate techniques was also conducted.

The techniques are used based on comparing the territorial division of Brazil (states) considering the following indicators: social difference (income and education data); public financial aid programs for the unemployed; and, the result of 2018 Presidential election identifying states where Bolsonaro received the most votes.

Thus, the objective of this study is to examine the Brazilian population during the period of the COVID-19 pandemic in Brazil with respect to research in infodemic terms, and to analyze possible behavior relating these investigations to the socioeconomic and political characteristics of the country.

## Materials & Methods

In this study, we retrieved metadata from seven free and open access information sources on the Internet, six of which are public databases with nationally consolidated data: Brazilian Institute of Geography and Statistics (Instituto Brasileiro de Geografia e Estatística (IBGE, 2021b)), National Telecommunications Agency (Painéis de Dados, 2021), Coronavirus Portal (Ministério da Saúde, 2021a), Transparency Portal (Portal da Transparência, 2021), Portal of the Labor Statistics Dissemination Program (PDET, 2020) and data from the Superior Electoral Court (Tribunal Superior Eleitoral, 2018). The other data source is GT (Google Trends, 2021b), a free web-based data source on trends in web information search in different geographic areas.

### Data extraction

Data extraction started by defining the search terms, which were chosen based on the works of Rovetta & Bhagavathula (2020a) and Rovetta & Bhagavathula (2020b) and later researched on the largest news sites in Brazil. The terms selected were the ones that stood out the most on these sites, in news and infodemic texts.

After this initial step, the search for terms in GT was started, capturing 18 months of information. First we used the methodology shown in Mavragani & Ochoa (2019) to understand the operation of the GT, and for each result topics and queries related were evaluated in order to reinforce the infodemic relevance of the term. For example, the query “covid food” may include searches for adequate nutrition during COVID-19 infection (*i.e.,* a non-infodemic topic), but we noticed that in the researched period there was a relationship with foods that treated the disease. Another example is the term “kill covid” referring to what could kill the virus, with results related to how chemical compound kills the virus, chlorine ingestion kills the virus.

The research in the GT was carried out in two stages. In the first one, the following GT filters were applied: geography, category and type of search that contained values such as “Brazil”, “all categories” and “web search”, and the second stage was carried out for each Brazilian state (27 states in total) keeping the options for “all categories” and “research on the Web”, and the collection took place on August 10, 2021. The GT returns the data in the form of Relative Search Volume (RSV) on a popularity scale: 0 (less popular) to 100 (very popular). The terms chosen were classified into five categories (Table 1).

**Table 1:** Infodemic terms by category. Thirty-one terms or term associations were selected to perform searches in the GT. Terms are grouped by categories.

**Categories**	**Terms**
Denomination	Coronavirus
	Covid
	Corona
	SARS
Origin	5G coronavirus
	Bill gates + bill gates virus
	Chinese virus
	China virus
Prevention and Beliefs	No masks
	No isolation
	Gargle
	Coronavirus gargle
	Garlic + garlic consumption + eating raw garlic is bad coronavirus garlic
	Kill covid
	Milk covid
Treatment	Chloroquine
	Covid chloroquine
	Coronavirus chloroquine
	Chloroquine
	Chloroquine trump
	Chloroquine china
	Ivermectin
	How to take ivermectin 6 mg
	Covid food
Vaccine	Alligator vaccine
	Doria vaccine
	Vaccine kills + dna vaccine
	Covid cancer + covid cancer vaccine
	Cause covid vaccine
	Alcoholic drink covid vaccine

Some terms that showed up during the pandemic within the context of “Vaccine” category are very typical for the Brazilian case: ‘alligator vaccine—the searches have grown after the President published, in December 2020, that those taking vaccine may become alligators; “doria vaccine”—after the President said that the vaccines did not to belong to the then Governor, João Doria. João Doria, unlike the President, adopted preventive measures following WHO guidelines right from the beginning of the pandemic. All the terms used in the investigation are in the Supplementary Material.

After the GT data, Internet access data available on the National Telecommunications Agency website (Painéis de Dados, 2021) were captured, such as fixed broadband, and mobile phone. These data inform how Internet accesses behave in each state.

From the portal of the Coronavirus Panel (Ministério da Saúde, 2021a), we use the spreadsheet that is made available daily with data from COVID-19, new number of cases per day and number of deaths per day, from all Brazilian cities.

To understand income and schooling inequality in Brazilian states, data from the country’s socioeconomic development were used. This database is provided by the PNADC, a survey that investigates the socioeconomic characteristics of the population (IBGE, 2021b).

The survey includes lots of information such as gender, age, education level, urban or rural housing, color or race, whether any course recently taken, whether the citizen is a partner of any company. To compose the database, 20 most significant variables were used for the research.

The downloaded information was treated with the PNADcIBGE package, developed by Braga (2017), with the R programming language, in the available version 3.6.3 (R Core Team, 2020).

Other important information for the research is data from government programs for cash transfers. The first program, in force since 1991, is unemployment insurance, which guarantees an income for 6 months for those who lost their jobs without justifiable cause. The data are available on the Labor Statistics Dissemination Program Portal (PDET, 2020). The second program, created in the pandemic, as an emergency, is the emergency aid, from April 2020 to October 2021, to help those who became unemployed during this period (Portal da Transparência, 2021).

To finalize the construction of the database for this work, voting results for the last presidential election, in 2018 were used. the decisive round there were two candidates: Fernando Haddad and Jair Bolsonaro, the last being elected. The data are from the Superior Electoral Court (Tribunal Superior Eleitoral, 2018).

### Data processing

The data processing stage used information from different sources. Since noise and distorted or null data could harm the analysis with incorrect information, each database was treated separately, limiting the characteristics of each base. After this first processing, the information was merged into a single database, adjusting by quarter and by state, thus facilitating the understanding of the data over the six quarters that were studied.

The grouping by data in quarter occurred due to the PNADC database making the data available every three months, totaling six quarters.

As Brazil is a very large country with many states with different population numbers, we performed the transformation of some variables by the rate that is proportional to one hundred thousand inhabitants (Eq. (1)), resulting in values proportional to the size of inhabitants of each state. The interpretation of the results would have a better understanding. Equation (1) displays *n* representing the quantitative of the variable, *pop* the number of the existing population in the state.

(1) \begin{eqnarray*}r= \frac{n}{\text{pop}} 100.000.\end{eqnarray*}

After this processing, the entire dataset was stored in a single database, containing all quarters. And for better monitoring and analysis of the evolution of the pandemic, the base was divided into quarters and the following nomenclature was used:

• 1st Q: Jan to Mar-2020;
• 2nd Q: Apr to Jun-2020;
• 3rd Q: Jul to Sept-2020;
• 4th Q: Oct to Dec-2020;
• 5th Q: Jan to Mar-2021;
• 6th Q: Apr to Jun-2021.

Some analyses were carried out at the national level, covering the entire period. In these cases, there was no division into pandemic quarters.

### Data analysis

This study performs a retrospective longitudinal infodemiological survey of COVID-19 in Brazil to analyze research trends on infodemic terms divided into five categories and analyze infodemic related to the country’s social and political scenarios, with exploratory and multivariate techniques based on clustering.

Specific data will be used for each analysis studied: in the trend analysis, the GTs infodemic RSVs will be used; in the exploratory analysis data from income, education and infodemic RSVs, then data from government programs and infodemic RSVs and finally, electoral data and infodemic RSVs. In the case of multivariate analysis, the database was used with information from the three scenarios, in addition to information on Internet access and numbers of deaths and cases of COVID-19.

We opted for unsupervised machine learning, with the cluster analysis method, because in multivariate analysis we use a dataset without labels or any kind of information about how the instances should be manipulated.

**Figure 1:**
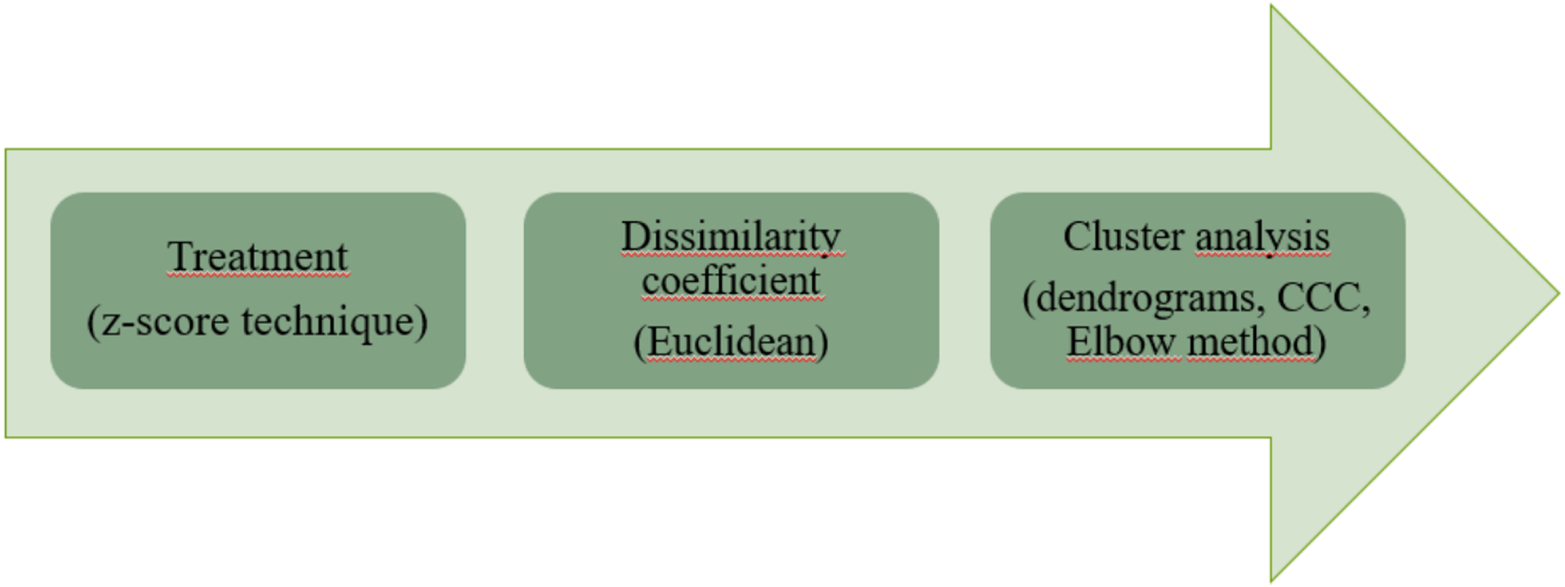
Clustering algorithm methodology. The steps are presented in the order of execution: data treatment with the *z*-score technique, dissimilarity coefficient with the chosen Euclidean method and the cluster analysis step, where the cophenetic correlation coefficient (CCC) was calculated, generated the dendrograms and the Elbow method was used in parallel with the dendrograms.

The structure of the cluster analysis application adopted is illustrated in Fig. 1.

Initially the data were in different units, and the grouping structure could change (Berthold et al., 2020). Thus, the z-score technique (Eq. (2)) was used, allowing the data to have similar weights for the next step. This technique was chosen because it is the most used data standardization technique (Fávero et al., 2009). The *z*-score technique standardizes each variable in such a way that the average is 0 and standard deviation is 1 leading to a same weight to each variable. Each attribute was standardized according to Eq. (1), where *X* represents the original value of the variable, µis the mean of the values associated with the variable, *σ* the standard deviation of the variable and *zX* the standardized value.

(2) \begin{eqnarray*}zX= \frac{X-\mu }{\sigma } .\end{eqnarray*}

After standardizing the data, it is necessary to define how similar the information is, to be grouped (Fávero et al., 2009). This calculation is defined by the similarity coefficient, or also dissimilarity, where the higher the value, the less similar are the instances. The choose was by the latter and by the Euclidean distance, because in some studies it is pointed out as being the measure of distance most used in cluster analysis. The Euclidean distance between the two patterns, *P*_*i*_ and *P*_*j*_, in an *m*-dimensional space is defined by Eq.

(3) \begin{eqnarray*}d \left( {P}_{i},{P}_{j} \right) =\sqrt{\sum _{k=1}^{m}({P}_{{i}_{k}}-{P}_{{j}_{k}})^{2}}.\end{eqnarray*}

Where *d*(*P*_*i*_, *P*_*j*_) is the distance between elements *P*_*i*_ and *P*_*j*_; *P*_*i*_*k*__ is the value of the indicator *P*_*i*_, for element *P*_*i*_; *P*_*j*_*k*__ isthe element value for the observation *P*_*j*_; the sum is performed for all indicators (*p*) considered.

There are many clustering methods, and choosing the process was the next step.

Methods such as ward, single, average, were used, in several tests, to identify the best results. The average method, in all interactions, showed the best results.

Equation 4 presents the formula of the average method where *Ci* and *Cj* are respectively the number of objects of the groups *C*_*i*_ and *C*_*j*_, and *x*_*a*_ and *x*_*b*_ are respectively the patterns of classes *C*_*i*_ and *C*_*j*_.

(4) \begin{eqnarray*}d \left( {C}_{i},{C}_{j} \right) = \frac{1}{ \left\vert {C}_{i} \right\vert {|}{C}_{j}{|}} \sum _{\begin{array}{@{}c@{}} \displaystyle {x}_{a}\in {C}_{i}\\ \displaystyle {x}_{b}\in {C}_{j} \end{array}}d \left( {x}_{a},{x}_{b} \right) .\end{eqnarray*}

For the graphic representation of the result of the grouping of brazilian states, the Dendrogram or Tree Diagram (Han, Kamber & Pei, 2012) was used. With the results of the dendrograms, the figures of the grouping of the Brazilian Regions and their respective states were created. In order to assess the degree of deformation caused by the construction of the dendrogram, we used the calculation of the cophenetic correlation coefficient (*CCC*) (Valentin, 2000; Sokal & Rohlf, 1962), which measures the degree of adjustment between the elaborated dendrogram and the dissimilarity matrix. Therefore, we chose to use the *CCC* in this study (Eq. (5)), where *F* represents the phenetic matrix and *C* depicts the cophenetic matrix.

(5) \begin{eqnarray*}CCC= \frac{C\hat {o}v(F,C)}{\sqrt{V \left( F \right) V(C)}} .\end{eqnarray*}

In this context of the CCC calculation, the higher the value of the result, the better the cluster adjustment, with the ideal being greater than 0.7 (Sokal & Rohlf, 1962).

The last step, used to corroborate the results of the dendrograms, was the Elbow method, of the non-hierarchical K-means clustering algorithm (Syakur et al., 2018), providing the ideal number of clusters. To calculate this method, the Sum of Squared Error is used, according to Syakur et al. (2018) (Eq. (6)), where k is the number of originated groups; *C*_*k*_ is the *i*-th cluster; *X* refers to the data in each group:

(6) \begin{eqnarray*}SSE=\sum _{k=1}^{k}\sum _{{X}_{j}\in {S}_{k}}{|}{X}_{j}-{C}_{k}{{|}}^{2}.\end{eqnarray*}

As the CCC and the Elbow method were used, both showing favorable results, the evaluation of outliers was not considered. The language chosen for data processing was Python, and Tableau (version 2021.3.3; https://www.tableau.com/support/releases/server/2021.3.3) was used for the visualization of results. The code was executed in the multivalued analysis step and for all pandemic quarters.

## Results

### Trends in search of infodemic terms in the Pandemic in Brazil

**Figure 2:**
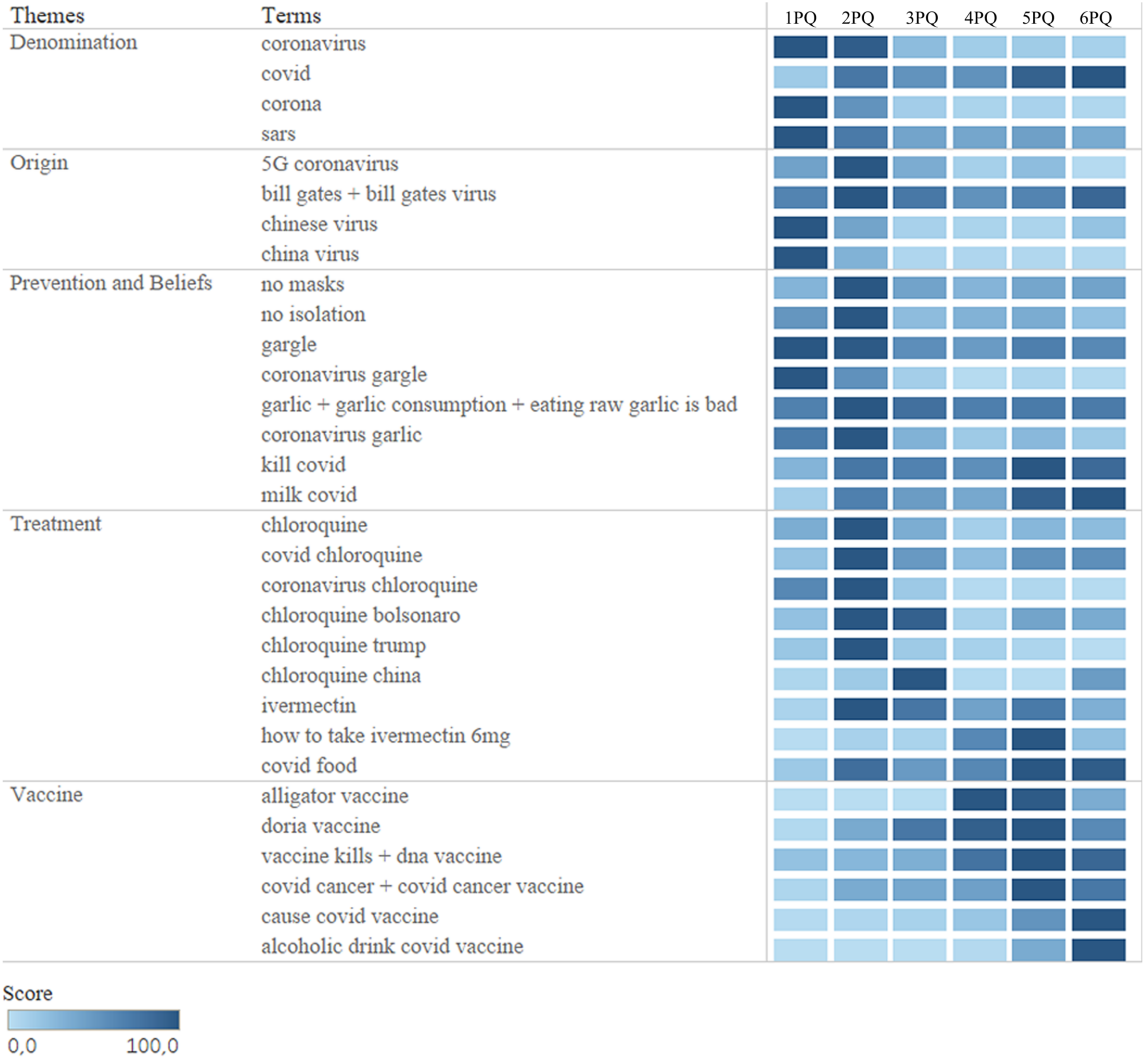
Infodemic searches in the GT in the period of 18 months. Eighteen months of research for all terms in the five categories studied, between January 1, 2020 and June 30, 2021, divided into six quarters.

We carried out a survey of this research trend in Brazil (Fig. 2), in the pandemic, over a period of 18 months, observing concrete changes in all categories. Some surveys maintained a pattern throughout the quarters and others more punctual. As the pandemic evolved and new information was introduced in the world and in Brazilian society, the searches were being updated.

The “Denomination” category is based on non-scientific names that are used for the SARS-CoV-2 virus that causes the disease COVID-19, with the term “covid” (the simplest form of writing and searching) with much more demand in the searches since April 2020. The other terms also appeared in searches, but in lower volume. This category was chosen because the terms searched for the name of the new coronavirus can be very generic and mislead to identify SARS-CoV-2, easily making the population confused with other coronaviruses (Rovetta & Castaldo, 2022).

The next category, “Origin”, are the terms that involve the appearance of the virus and were considered the highest values in the searches linking the virus to businessman Bill Gates, as mentioned in Duarte (2020) in his interview (in 2015) about the emergence of a possible pandemic in the near future.

The following two categories had higher numbers in the RSVs. Highlighting the words “not masks” and “in isolation”. They gained national importance after the president failed to respect WHO protocols by issuing a statement that “the correct use of masks brings zero efficiency” (Aos Fatos, 2021).

Finally, the latter category had the terms related to dying after taking the vaccine or acquiring COVID-19 after the vaccine as the most researched in 2021, just when vaccination began, which occurred in the first month of 2021.

### Analysis of the country’s social and political scenarios: exploratory and multivariate techniques based on clustering

In order to have a better analysis of the data studied, techniques were used on the variables highlighted for each scenario. The exploratory analysis was presented in bar graphs allowing a better understanding of the data studied, in the period between January 1, 2020 and June 30, 2021.

### Social inequality: per capita income and schooling

**Figure 3:**
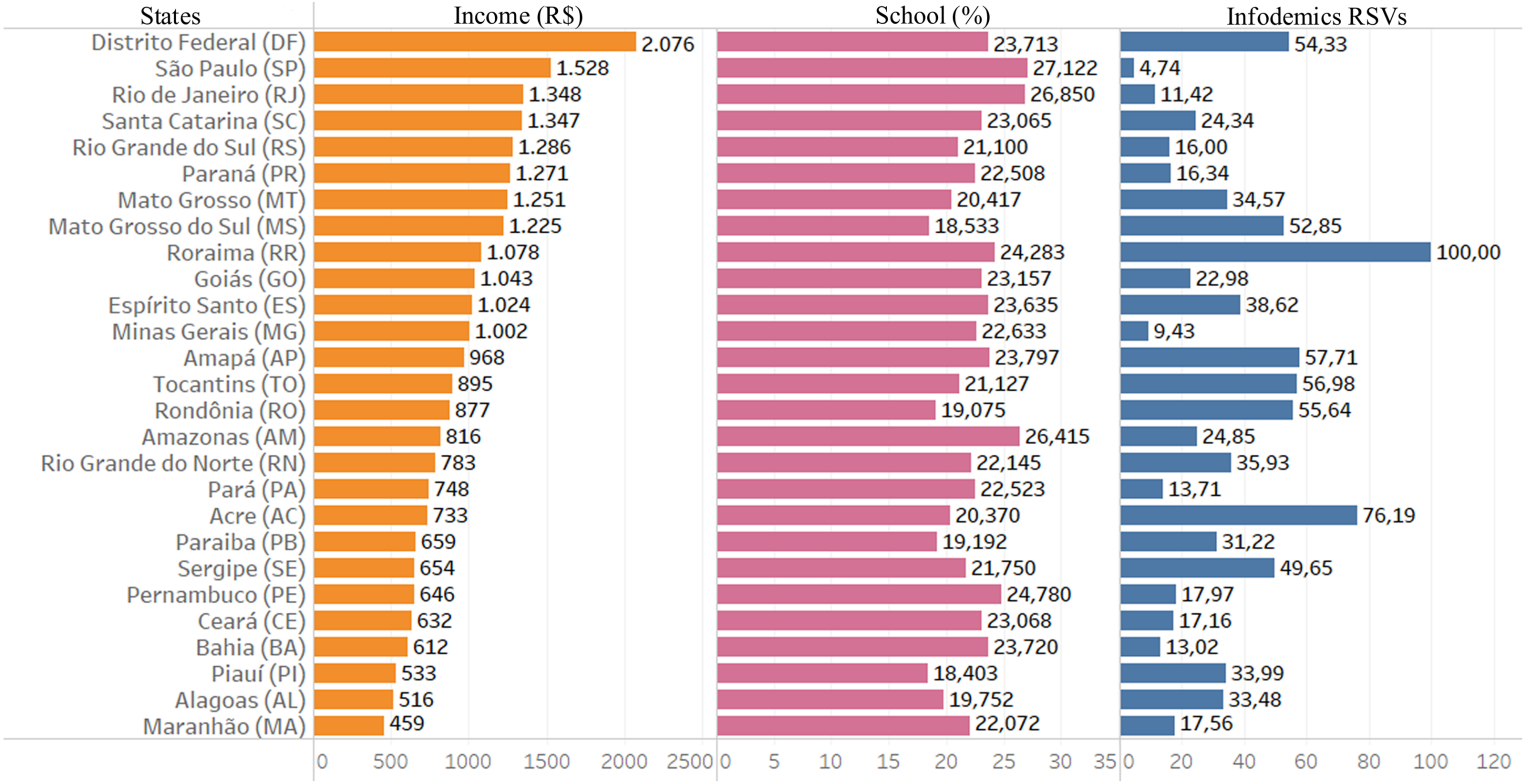
Data from infodemics RSVs, income and education. The columns represent the 18-month average of information for each Brazilian state: The first column is the average per capita income in Brazilian currency; the second column represents the average, in percentage, of the level of education in completed secondary education and the last column the average of infodemics RSVs.

Figure 3 presents the information in the form of bar graph of Brazil in infodemic survey data on the COVID-19 pandemic, in addition to socioeconomic variables such as average per capita income and high school level, showing the social reality of each state.

Looking at Fig. 3, it is possible to observe a very large income inequality, ranging between R$ 2,076 and R$ 459, the highest in the DF and the lowest in the state of MA.

It is possible to visualize and perceive information from similar indexes of infodemic searches in states where there are variations between income, as in DF and SE, and also PA and MG, and great variations in schooling (DF and MS). There is no infodemic search pattern based on income or education.

**Figure 4:**
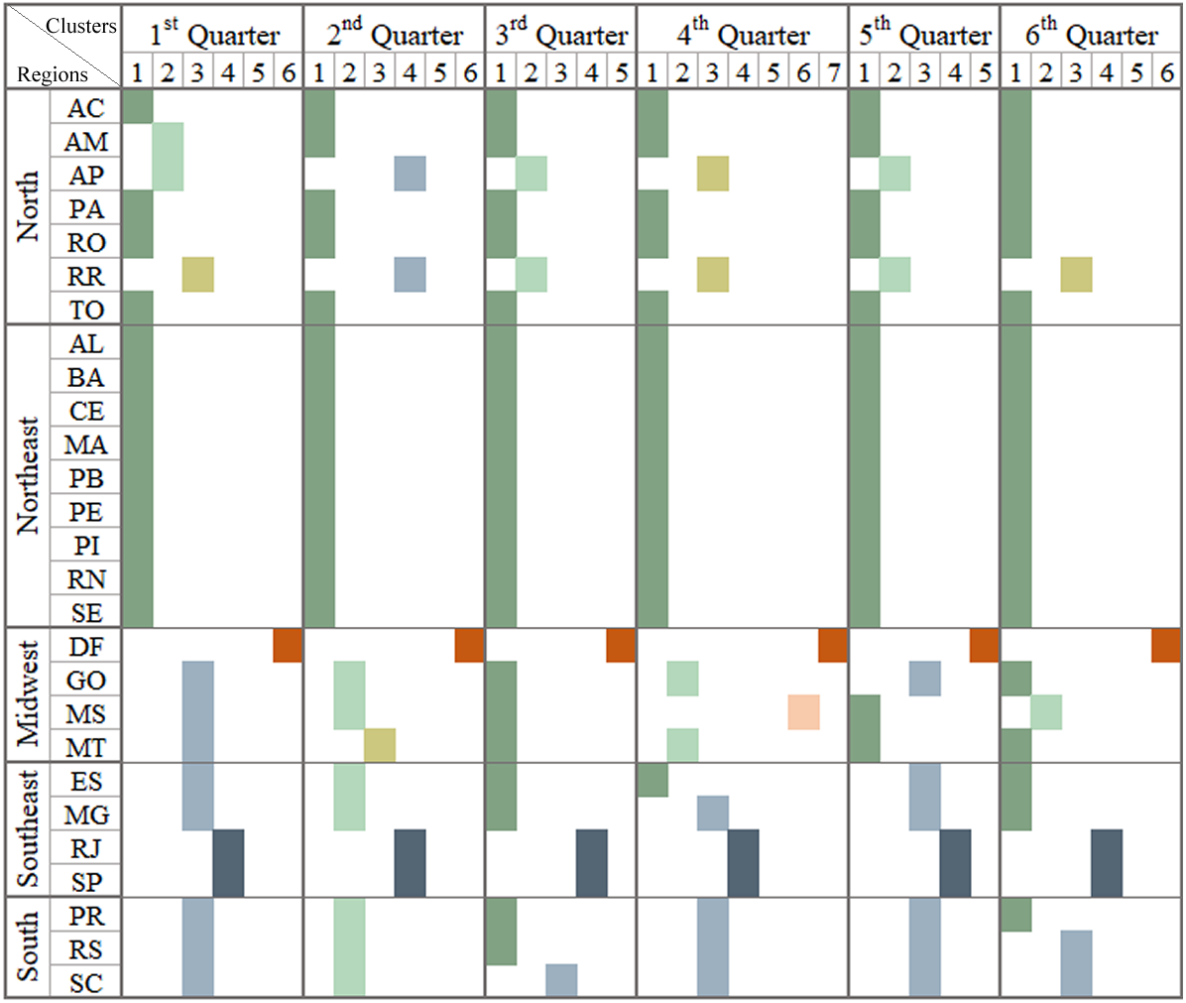
Brazilian regions grouped by infodemic searches and social differences. The multivalued analysis technique was applied in each quarter, grouping Brazil into a different format of the Brazilian regions: what can be observed in each column representing a quarter. It presents satisfactory CCC (0.89, 0.88, 0.91, 0.90, 0.87 and 0.91, respectively, for each pandemic quarter) and the number of clusters was 6, 6, 5, 7, 5 and 6.

As for the multivariate analysis, we used, in addition to the variables in Fig. 3, Internet access data, population research trends in infodemic terms and numbers related to COVID-19 (deaths and case numbers), to identify events in the evolution of the pandemic. and attitudes of the population. Each column in the image represents similar patterns of behavior regarding information on: per capita income variables, high school education level, infodemic research, Internet access and numbers related to COVID-19.

The first three columns in Fig. 4 show the 1st Q, 2nd Q and 3rd Q, from January to September 2020, covering the first wave of the pandemic in Brazil, and it was observed that in the 1st and 2nd Q 6 clusters were generated and in the next five clusters, with different numbers of states in each group. The values of 0.89, 0.88 and 0.91 for CCCs for the generated dendrograms were considered satisfactory when comparting with the dissimilarity matrix.

Different regions of Brazil were affected in different manner and this can be observed in the clusters shown in Fig. 4. Based on some reported cases, the pandemic occurred first in SP and then in RJ and within two weeks, other states in other regions of Southeast, Northeast, Midwest, and South also started reporting cases of the disease. North became an epicenter at the beginning of the second quarter of 2020. The groupings of the 2nd quarter began with equivalent characteristics when the population received numerous information, many incorrect, even from reliable sources.

The northeast region was hit by an increase in the number of cases by mid-June. Already evaluating the central region of Brazil (Midwest), which was slow to have significant numbers of deaths and cases of COVID-19, only in the 3rd quarter, showed a significant increase in cases, followed by the most extreme region (South) (Mota & Teixeira, 2020). In Fig. 4, it is possible to infer that the populations looking for infodemic information doesn’t have any relation whether the state’s income and education levels are high or not. But it was related to the increase in the number of cases and deaths due to the pandemic.

Observing the clusters displayed in the last three columns of Fig. 4, the analysis pointed out the states with similar behaviors being SP and RJ and AP and RR, and DF forming an isolated group in all dendrograms.

At the end of the 3rd quarter, there was influence, of a descending curve of the first wave, on the cluster patterns for the 4th Q, with the maximum number of clusters, so far. In October there were elections in the Brazilian states diverting the infodemic searches for searches related to the elections. In the last month of 2020, new variants, Delta and Gama (first identified in the AM capital), were announced modifying the scenarios in the 5th and 6th quarters. In January 2021, there was an increasing number of cases and deaths in Brazilian regions, leading to an even worse scenario than in 2020, covering up to the second wave.

### Social income transfer programs

**Figure 5:**
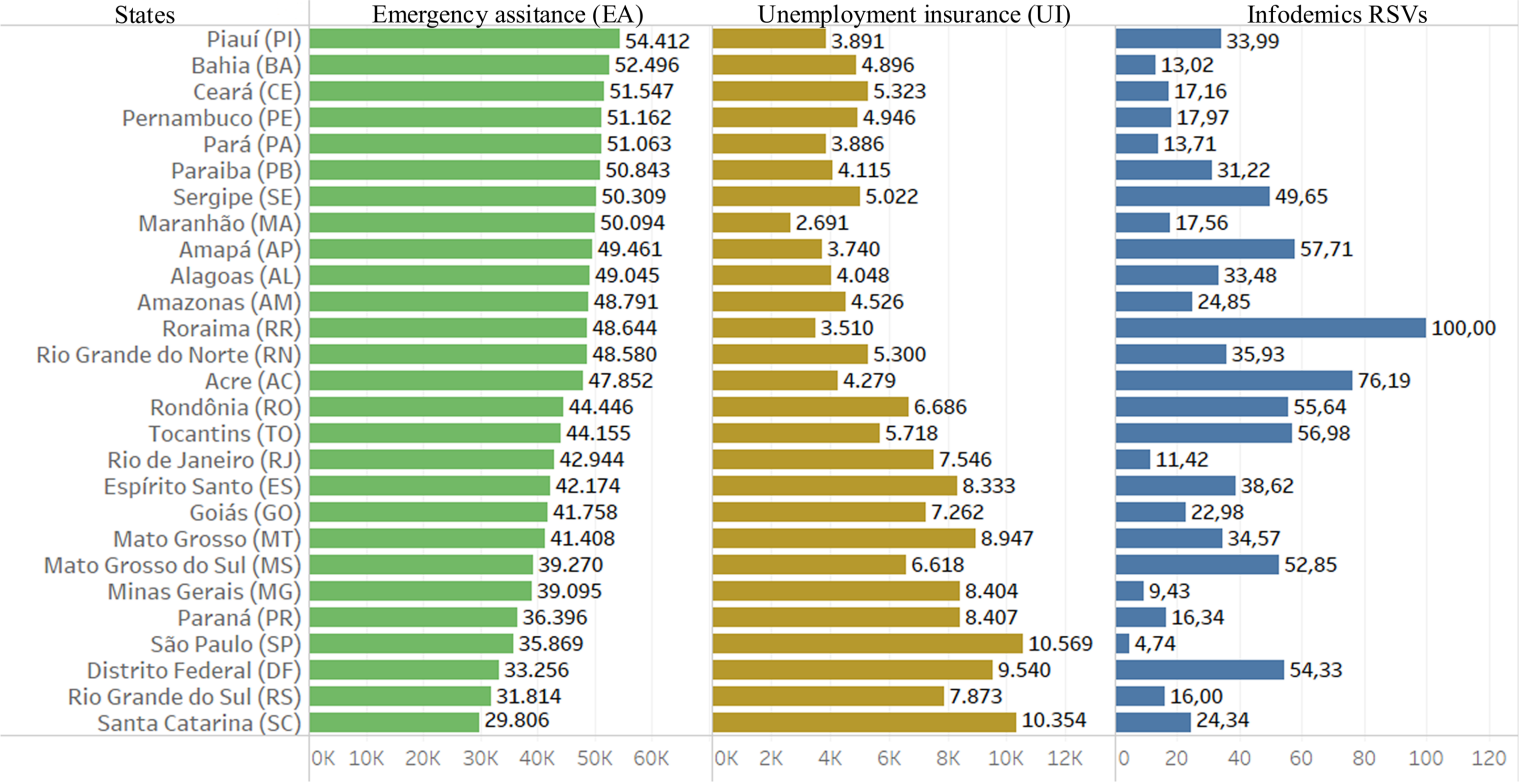
Data from infodemic RSVs and income distribution programs. The columns represent the average of 18 months of information for each Brazilian state: the first column is the average payment of emergency aid in local currency; the second column represents the average number of unemployment insurance claims and the last column the average of RSVs infodemics.

Another part of the work is to analyze the Brazilian government’s income transfer programs used in the pandemic. In Fig. 5 one can see data from searches for infodemic terms, rates of emergency assistance payments (started in April 2020) and unemployment insurance claims. We verified on the website that Q1 2020 saw an increase of almost 20% compared to Q4 2019, and an increase of almost 125% from Q2 to Q1 2020.

Far from being homogeneous, Brazilian states portray inequality in requesting assistance to the government programs. The North and Northeast states applied mostly for emergency assistance, while the regions of South, Southeast and Midwest mostly for unemployment insurance.

**Figure 6:**
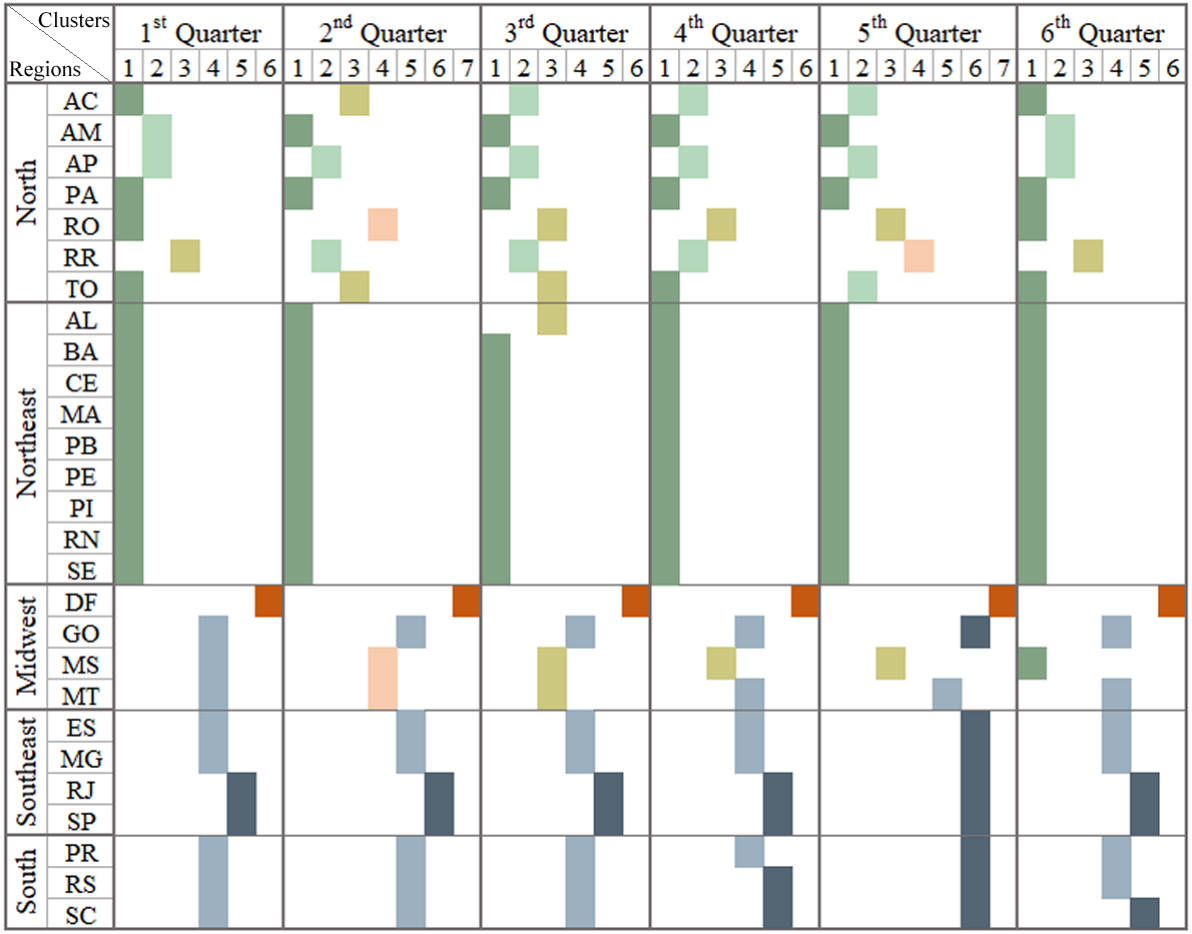
Brazilian regions grouped by infodemic searches and money transfer programs. The multivalued analysis technique was applied in each quarter, grouping Brazil into a different format of the Brazilian regions: what can be observed in each column representing a quarter. It presents satisfactory CCC (0.82, 0.78, 0.78, 0.82, 0.80 and 0.77, respectively, for each pandemic quarter) and the number of clusters was six, seven, six, six, seven and six.

As for the cluster analysis technique, two variables were added to the survey, unemployment insurance numbers and emergency assistance numbers. The results of clustering are shown in Fig. 6, where each column in the image represents similar patterns of behavior in relation to information from the multivalued analysis of Fig. 4 plus data on emergency aid and unemployment insurance.

The columns refer to groups of standards similar to government actions for the unemployed to have an income during a certain period (Costa, 2020). From the 2nd Q until the 5th Q, one can observe that some regions had similar patterns, such as States in North, Northeast and South.

The COVID-19 pandemic strongly affected the labor force leading to high unemployment in Brazil (Barros, 2021). The northeastern states were the most affected and the least affected in the south. The unemployment rate was record in 2020 affecting 20 states increasing the national average 1.6% between 2019 and 2021 (Barros, 2021).

There was also a record of freelancer. Seven out of 10 jobs created were independent (Alvarenga & Silveira, 2021) and may have contributed to the number of clusters generated in 2020 and 2021.

Looking at the last quarter, there is already a fall in the states by clusters, corresponding to a small increase in employment in Brazil (IBGE, 2021a).

There was also a record self-employment. Seven out of 10 jobs created were independent (Alvarenga & Silveira, 2021) which may have influenced the number of clusters in 2020 and 2021.

Looking at the last quarter, there is already a fall in the states by clusters, corresponding to a small increase in employment in Brazil (IBGE, 2021a).

### Political influences: president supporters

Leaders of the countries and their attitudes and actions influence the countries and also can affect the beliefs and behaviors of the populations (Dewan & Myatt, 2008). As elsewhere mentioned, in Brazil the second round of elections of 2018 had two candidates: Jair Bolsonaro and Fernando Haddad. The elected president was Jair Bolsonaro.

**Figure 7:**
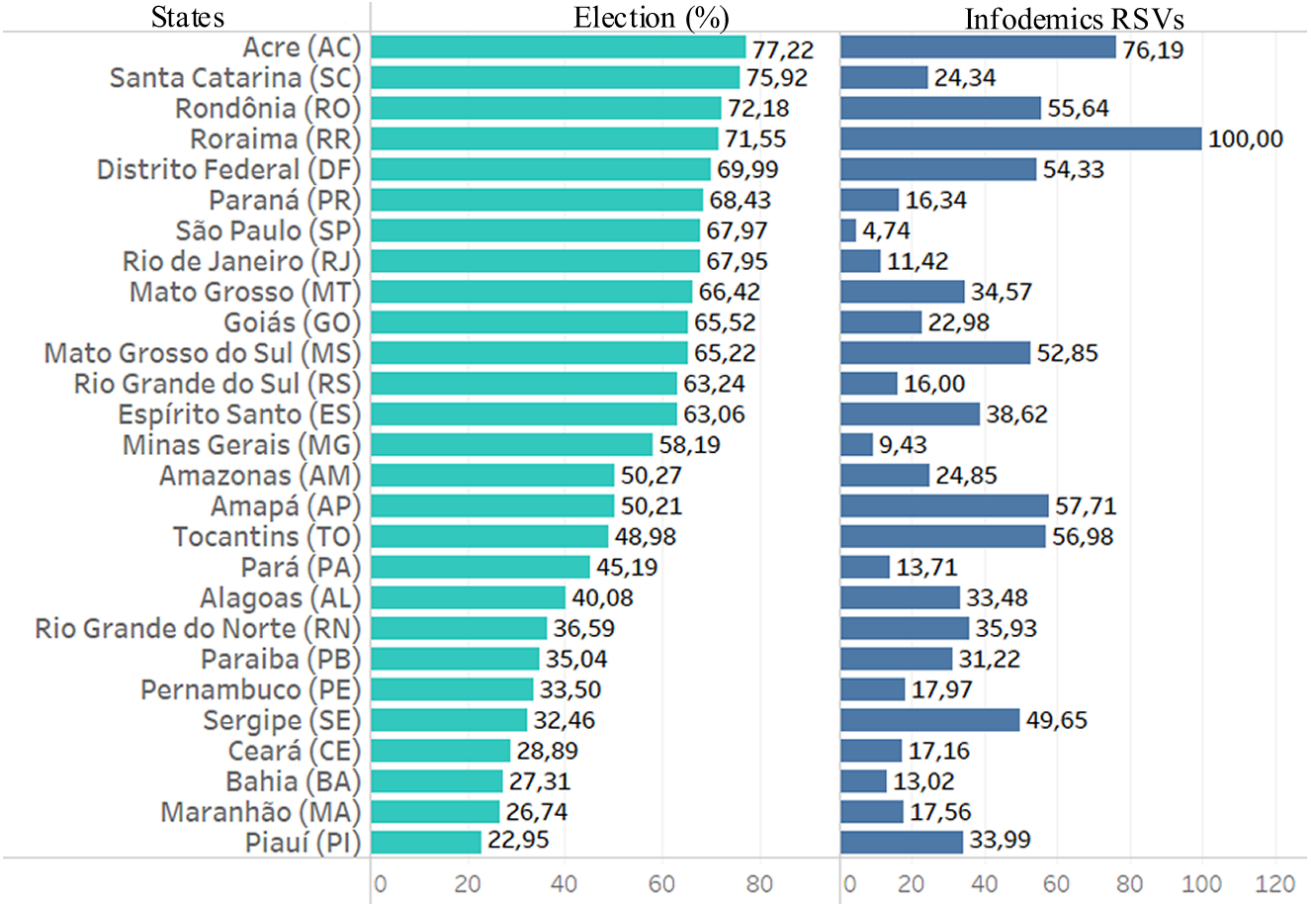
Data from infodemic RSVs and 2018 elections. The first column represents the percentage of votes from each state for the elected president, and the last column represents the average of the RSVs infodemics.

The bar graph in Fig. 7 illustrates each state’s percentage of votes for the president-elect. It can be seen that in 16 states this percentage increased to 50%. Interestingly, populations in these states searched more for infodemic terms. These states presented RSVs data with a value of 40% more than in the other 11 states.

**Figure 8:**
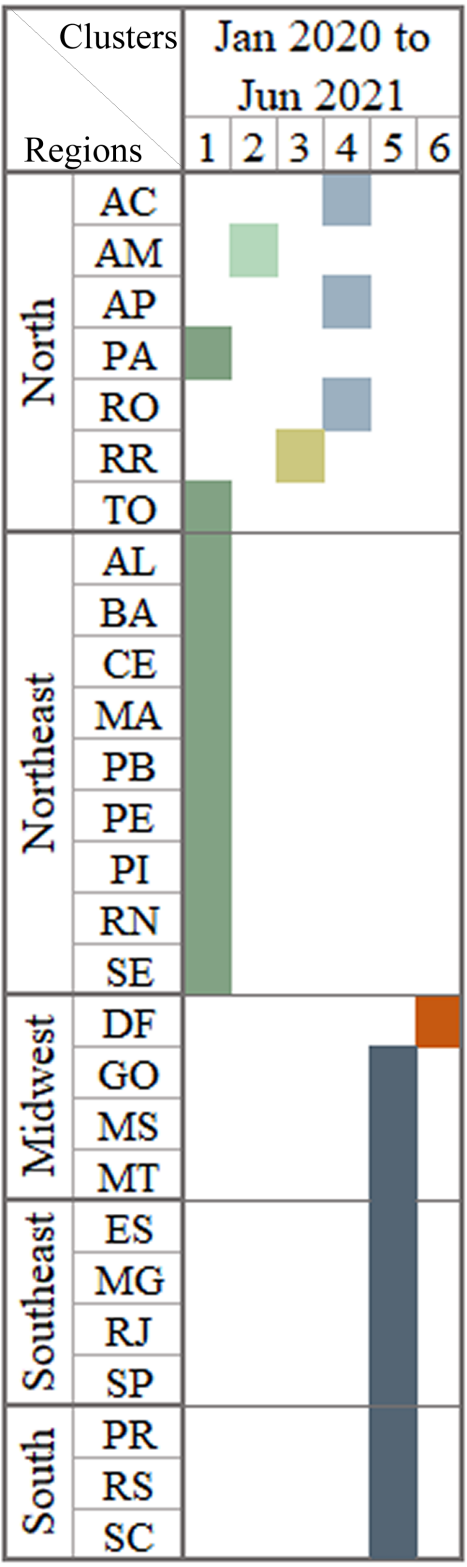
Brazilian regions grouped by infodemic searches and 2018 elections. The multivalued analysis technique was applied throughout the period, generating clusters presented in the column with satisfactory CCC (0.86), and having the number 6 clusters.

The last step was to perform the cluster technique on the basis of election data and 18-month national data from infodemic surveys. No more for data divided into Brazilian states. Figure 8 shows the columns with the generated clusters and presented a result of 0.86 for the CCC coefficient. The cluster one is formatted by states where the president received less than half of the votes and presented numbers with similar patterns of infodemic polls that were not much influenced by the political leader.

The columns represents similar patterns of behavior using the information already presented in Fig. 6 plus the percentage of votes in the 2018 elections.

## Discussion

It is understood that of all the categories surveyed those that emerged in the researches were those of content that most interfere with the Brazilian culture: “prevention and beliefs” and “treatment”. Most of the Brazilians believe in treatments that are either ineffective or without scientific evidence. Unfortunately, this portion of the population never gave due importance to the pandemic, did not believe that vaccines were effective despite the political authorities’ encouragement.

For example, in India at the end of 2021, the Medical Council banned chloroquine and ivermectin to treat COVID-19. Unlike Brazil, which was among the first countries in pandemic numbers, it persisted that such treatments would be effective. One of the president’s statements was that if he became infected with SARS-CoV-2, he would begin treatment with the so-called “covid kit” and such statements are somehow part of the population believed to be true.

A more detailed query was carried out for the terms “coronavirus” or “corona” in the GT, where between the years 2017 and 2019, it resulted in few RSV queries. And to the term “sars”, only 69 RSVs. These are very small numbers, since the terms had already been known for two decades.

In some countries, for example, United Kingdom, there were searches for the term “5G coronavirus”, but not that much in Brazil. In this country there was a substantial search on a social network in the hashtag #5GCoronavirus (Ahmed et al., 2020). At the time, Brazil did not have antennas with 5G signal and therefore it is understandable that not many were looking into this term. Terms “chinese virus” or “china virus” were also not searched for in Brazil as in other countries (Budhwani & Sun, 2020; Hu et al., 2020).

The quarters showed searches on preventing the disease by means of some foods as well as gargling. One such featured search was “garlic+garlic consumption+garlic consumption is bad”. This combination is something quite common in Brazil, but increased a lot in searching for food that could fight the disease. Another term ’alligator vaccine’ was thoroughly researched, after the President’s intervention in December 2020, mocking the vaccines by commenting that “it has no result”, in addition to stating that the vaccine can turn person into alligator (Silva, 2021). Thus, ‘alligator vaccine’ was heavily searched.

The search for infodemic information was the highlight from the analysis with exploratory and multivariate techniques. This search was permanent in the months surveyed. Many variables were used to identify and understand the impacts on search patterns and observed that:

• States with high per capita income and states with low per capita income had similar numbers of infodemic surveys. Comparing the data from the two extreme income states, the search for infodemics was more than triple in DF compared to MA, with no pattern in these searches. There are also no patterns in infodemic searches for states with similar percentages of schooling.
• After analyzing the first three quarters, SP and RJ, AP and RR and the DF formed a single group in all the dendrograms. This occurred as the similarity of these states in terms of behavior and characteristics is quite close for the observed period and different from other states.
• The results analyzed in the last three quarters clearly place the DF in an isolated cluster. RJ and SP form a single cluster in addition to pointing out that RR is always in a single cluster, or shares with AP. The state of RR is marked by the high income factor, which impacts on their groupings. It also has high rates in other variables and remains constant in the results.
• The analysis conducted after the grouping indicates that states with high or low monthly income illustrate similar patterns.

The research evaluated how income aid programs funded by the Federal Government could influence research on false information about the pandemic. Since the beginning of the pandemic, searches for these types of aid programs have increased substantially due to the high number of unemployment, as the population needed assistance, and yet searches for pandemic infodemic terms also continued to grow. What may have occurred due to the various failures of governments to combat fake news, lack of adequate control measures and WHO guidelines. Looking at Fig. 6, some states remained grouped in almost all quarters, states located in regions with lower income, such as the Northeast and North regions.

Health professionals and scientists have worked and are still working hard to control and prevent the pandemic, following WHO guidelines and disseminating quality information, but many authorities acted in the opposite way, greatly harming the fight against the pandemic. What can be seen in the last scenario, which identified how the influence of the president impacted the search for infodemic terms in the states where the president obtained more than 50% of the votes, were the states that most searched for infodemic terms.

Other observations are relevant, such as: the AM and DF states formed a separate group based on multivariate analysis. These are the states, which have the highest and lowest values of Internet access. The regions with the highest monthly income values formed a single cluster. Other clusters were left with states with similar behavior in infodemic searches.

## Conclusions

Brazil presented negative results regarding the COVID-19 pandemic in regard to the frightening numbers of deaths and cases. We can cite some factors that aggravated these numbers, such as: changes in the highest authority of health, without affirmative actions. At the national level, lack of credibility in science and in the vaccine, contradictory and inaccurate information about preventive measures, and while the patient has the COVID-19, disease medication (called a “covid kit”), denial affected the country’s scenario in a negative way. As seen in this research, and in the techniques used, these factors acted to make Brazil totally unprepared for a pandemic and constantly looking for infodemic information.

The infodemic, the central study of this research, is already considered a worldwide fear, already presented at the World Economic Congress almost ten years ago (World Economic Forum, 2013). It is important to outline strategies and public policy actions to combat the infodemic, and in this sense it was crucial to carry out cluster analysis to group states with similar patterns. It is important to enable the understanding of the population’s reaction to events such as failures in the Federal Government to face the pandemic and, thus, infer the reason for the excessive infodemic searches.

Future studies are in planning stage by extending the database to integrate two years of infodemic RSVs data and focusing more in terms of vaccination, and number of vaccinated in Brazil, by state, and by region. In this aspect, data by age group, comorbidities, healthcare professionals, and education professionals will be included. This should provide more scenarios of the behavior of the Brazilian population to assist policymakers and managers in planning and controlling health resources, preventing outbreaks and preventing the spread of the infodemic.

Some limitations are considered in this work. First, there is no guarantee of having included all the relevant keywords, since much infodemic information circulated on the Internet and the work was limited on some websites and papers, not from searching on social networks such as Facebook and Instagram. Another limitation is that the GT data are in relative research volume and not in absolute search volumes, which could lead to more accurate studies. However, the analysis of the data studied for the pandemic period brought useful information. Internet use is also a limitation, as there are percentage differences in Internet use in Brazilian states due to the number of different inhabitants per state and the physical access structure between regions. Another point to consider about the Internet is that its use can be age-dependent.

##  Supplemental Information

10.7717/peerj.13747/supp-1 Supplemental Information 1 Keyword selection Thirty-one terms or term associations were selected to perform searches in the GT. Terms are grouped by categories.

## Additional Information and Declarations

### Data Availability

The following information was supplied regarding data availability: The data is available at GitHub: https://github.com/mpenhaharb/BaseCodeGT.
