# Supplementary material for: The COVID-19 infodemic in Brazil: trends in Google search data
Source: PeerJ. 2022 Aug 4;10:e13747. doi: 10.7717/peerj.13747 (PMC9357377; doi:10.7717/peerj.13747)
Supplement: Supplemental Information 1 — Thirty-one terms or term associations were selected to perform searches in the GT. Terms are grouped by categories. [file peerj-10-13747-s001.pdf]

Keywords selection

The keywords used in Google Trends searches from January 1, 2020 to June 30, 2021 are listed below, by category.

- Category denomination:
  - coronavirus
  - covid
  - corona
  - SARS
- Category origin:
  - 5G coronavirus
  - bill gates + bill gates virus
  - chinese virus
  - china virus
- Category prevention and beliefs:
  - no masks
  - no isolation
  - gargle
  - coronavirus gargle
  - garlic + garlic consumption + eating raw garlic is bad
  - coronavirus garlic, kill covid, milk covid
- Category treatment:
  - chloroquine
  - covid chloroquine
  - coronavirus chloroquine
  - chloroquine
  - chloroquine trump
  - chloroquine china
  - ivermectin
  - how to take ivermectin 6mg
  - covid food
- Category vaccine:
  - alligator vaccine
  - doria vaccine
  - vaccine kills + dna vaccine, covid cancer + covid cancer vaccine
  - cause covid vaccine
  - alcoholic drink covid vaccine
